# Establishment of Epidemiological Cut-Off Values and the Distribution of Resistance Genes in *Aeromonas hydrophila* and *Aeromonas veronii* Isolated from Aquatic Animals

**DOI:** 10.3390/antibiotics11030343

**Published:** 2022-03-05

**Authors:** Soo-Ji Woo, Myoung-Sug Kim, Min-Gyeong Jeong, Mi-Young Do, Sung-Don Hwang, Woo-Jin Kim

**Affiliations:** 1 Aquaculture Industry Research Division, East Sea Fisheries Research Institute, National Institute of Fisheries Science, Gangneung 25435, Korea; sdhwang@korea.kr (S.-D.H.); wj2464@korea.kr (W.-J.K.); 2 Pathology Research Division, National Institute of Fisheries Science, Busan 46083, Korea; fishdoc@korea.kr (M.-S.K.); susymg@naver.com (M.-G.J.); domiyeong@gmail.com (M.-Y.D.)

**Keywords:** *Aeromonas* spp., antimicrobial resistance gene, ECOFFinder, epidemiological cut-off values, normalized resistance interpretation, MIC

## Abstract

The emergence of antimicrobial-resistant bacteria is an enormous challenge to public health. *Aeromonas hydrophila* and *Aeromonas veronii* are opportunistic pathogens in fish. They exert tremendous adverse effects on aquaculture production, owing to their acquired antibiotic resistance. A few Clinical and Laboratory Standards Institute (CLSI) epidemiological cut-off values (ECVs) against *Aeromonas* spp. are available. We evaluated antimicrobial susceptibility by establishing 8 ECVs using two analytical methods, normalized resistance interpretation and ECOFFinder. We detected antimicrobial resistance genes in two motile *Aeromonas* spp. isolated from aquatic animals. Results showed that 89.2% of *A. hydrophila* and 75.8% of *A. veronii* isolates were non-wild types according to the oxytetracycline ECV_CLSI_ and ECV_NRI_, respectively. The antimicrobial resistance genes included *tetA*, *tetB*, *tetD*, *tetE*, *cat*, *floR*, *qnrA*, *qnrB*, *qnrS*, *strA-strB*, and *aac(6′)-1b*. The most common *tet* gene in *Aeromonas* spp. isolates was *tetE*, followed by *tetA*. Some strains carried more than one *tet* gene, with *tetA*–*tetD* and *tetA*–*tetE* found in *A. hydrophila*; however, *tetB* was not detected in any of the strains. Furthermore, 18.6% of *A. hydrophila* and 24.2% of *A. veronii* isolates showed presumptive multidrug-resistant phenotypes. The emergence of multidrug resistance among aquatic aeromonads suggests the spread of drug resistance and difficult to treat bacterial infections.

## 1. Introduction

The genus *Aeromonas* comprises 36 species representing ubiquitous bacteria isolated from food, animal, and aquatic environments [1]. Among the salmonids, the genus *Aeromonas* is an enteric pathogen, which causes haemorrhagic septicaemia, fin rot, and soft-tissue rot companied by high mortality [2,3]. *Aeromonas* spp. produce a variety of toxins, including hemolysins, aerolysins, and cytotonic enterotoxins, which cause diarrhea, enteritis, and dysentery [4,5]. *Aeromonas* spp. are opportunistic bacteria commonly present in freshwater and marine environments, with *Aeromonas salmonicida* subsp. *salmonicida*, *Aeromonas hydrophila*, and *Aeromonas veronii* identified as causative agents of hemorrhagic skin ulcers and furunculosis in Nile tilapia, common carp, and channel catfish [1,6,7,8,9]. Pathogenic *Aeromonas* spp. kills 80–100% of commercial carp within 1–2 weeks, resulting in the deterioration of production quality in fisheries [10]. The resulting unfavorable conditions, such as hypoxia or nitrogen-waste accumulation, induce a significant reduction in immune response leading to increased risk of pathogen translocation, infection, and disease [11]. β-lactam-, aminoglycoside-, and quinolone-resistant strains of *Aeromonas* spp. have been isolated from water and fish worldwide [12,13,14]. Resistant strains have been isolated even from heavily polluted water; they harbor multiple resistant plasmids [15]. *Aeromonas* spp. can receive and deliver a set of gene-associated plasmids, integrons, and transposons [16]. These mobile elements are important for the delivery of genetic material and can specifically encode antimicrobial resistance. *Aeromonas* spp. resistant to several antimicrobials raises the issue of the One Health concept, which involves transmission of resistant pathogens to humans who share an aquatic source through the food chain or direct contact. Therefore, it is necessary to monitor the emergence of antimicrobial resistance in *Aeromonas* spp. to guide clinical treatment.

There is no effective vaccination against *Aeromonas* spp., because of the presence of various serotypes. Most infections caused by *Aeromonas* are treated using antimicrobial therapy. Another challenge in treating *Aeromonas* infections is the absence of Clinical and Laboratory Standards Institute (CLSI) antimicrobial breakpoints and susceptibility test protocols against *Aeromonas* spp., except those established for *A. salmonicida* [17]. Recently, the CLSI guideline (VET 04) updated the epidemiological cut-off values (ECVs) for the isolates of *A. salmonicida*, *A. hydrophila*, *Flavobacterium columnare*, and *F. psychrophilum* [18]. The ECV for *A. salmonicida* was established more than 10 years ago, and the isolates used to establish the ECV were not from fish that were part of a clinical field trial. The antimicrobial susceptibility of *Aeromonas* isolates have been extensively studied [19,20]; however, there are only a few studies, which determined the ECVs of *Aeromonas* spp. isolates from rivers and fish [21,22]. The antimicrobial susceptibilities of motile *Aeromonas* spp. isolates were determined by applying the florfenicol, tetracycline, and sulphonamide ECVs [23]. Therefore, it is necessary to evaluate antimicrobial-sensitivity data and ascertain the latest ECVs and resistance genes for pathogenic aquatic aeromonads sampled from the aquaculture field.

In this study, we determined the minimum inhibitory concentration (MIC) distributions, ECVs, and resistance genes for two representatives motile *Aeromonas* spp. (*A. hydrophila* and *A. veronii*) to demonstrate the possible hazards of excessive antimicrobial use in aquaculture, for both humans and animals.

## 2. Results

### 2.1. Antimicrobial Susceptibility

Distribution of the MICs for eight antimicrobial agents and the corresponding MIC_50_ and MIC_90_ against *A. hydrophila* and *A. veronii* were evaluated (Table 1 and Table 2). The MICs obtained for *Aeromonas* spp. isolates ranged from 0.25–64 µg mL^−1^ for doxycycline, 0.03–32 µg mL^−1^ for enrofloxacin, and 0.03–64 µg mL^−1^ for erythromycin and florfenicol. Among the antimicrobials, oxytetracycline had the highest MICs at >256 µg mL^−1^ for four *A. hydrophila* isolates and one *A. veronii* isolate. In *A. hydrophila*, differences between the MIC_50_ and MIC_90_ for flumequine, neomycin, and oxytetracycline were within two dilution steps; for florfenicol and enrofloxacin, five and six dilution steps, respectively. In *A. veronii*, differences between the MIC_50_ and MIC_90_ for gentamicin, neomycin, and oxytetracycline were within one dilution step; for florfenicol and flumequine, five and six dilution steps, respectively.

### 2.2. ECV Establishment Using Two Analytical Methods

**Figure 1:**
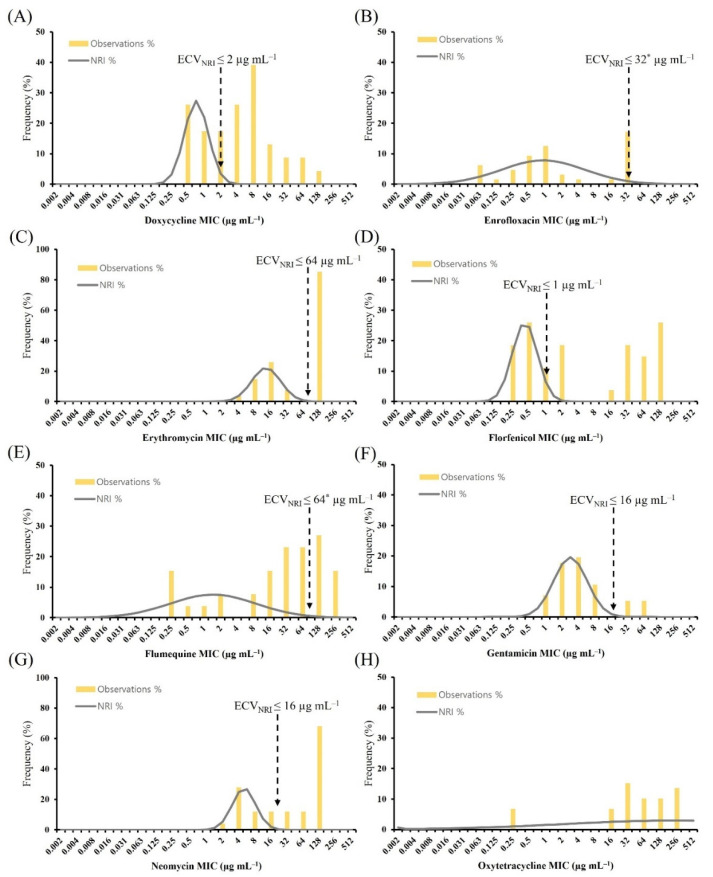
Distribution of MICs for *Aeromonas hydrophila*. MICs for *A. hydrophila* (*n* = 43) were determined using the broth microdilution method for (**A**) doxycycline, (**B**) enrofloxacin, (**C**) erythromycin, (**D**) florfenicol, (**E**) flumequine, (**F**) gentamicin, (**G**) neomycin, and (**H**) oxytetracycline. Gray line indicates the NRI-derived normal distribution of WT isolates. Yellow vertical lines indicate the ECVs calculated from the data. Vertical black dashed lines indicate the ECV_NRI_ determined in this study. The standard deviations for enrofloxacin and flumequine were >1.2 log_2_ (*). Oxytetracycline did not allow for ECV_NRI_ calculation. ECV, epidemiological cut-off value; MIC, minimum inhibitory concentration; NRI, normalized resistance interpretation; WT, wild type.

We aimed to establish the ECVs for doxycycline, enrofloxacin, erythromycin, florfenicol, flumequine, gentamicin, neomycin, and oxytetracycline by testing 43 *A. hydrophila* and 33 *A. veronii* isolates from various diseased aquatic animals using the normalized resistance interpretation (NRI) and ECOFFinder methods. Figure 1 shows the histogram of MICs for eight antimicrobial agents against *A. hydrophila* using the NRI method. Based on the MIC distributions, the ECV_NRI_ for doxycycline was 2 µg mL^−1^. This categorized 23 (53.5%) isolates as non-wild type (NWT); they exhibited reduced susceptibility. The ECV_NRI_ values for erythromycin and florfenicol were 64 µg mL^−1^ and 1 µg mL ^−1^, which categorized 23 (53.5%) isolates and 24 (55.8%) isolates as NWT, respectively. The ECV_NRI_ values for enrofloxacin and flumequine were 32 µg mL^−1^ and 64 µg mL^−1^, respectively; however, the standard deviation values of 1.2 log_2_ indicated inadequate precision. The NRI calculations did not generate results for oxytetracycline.

**Figure 2:**
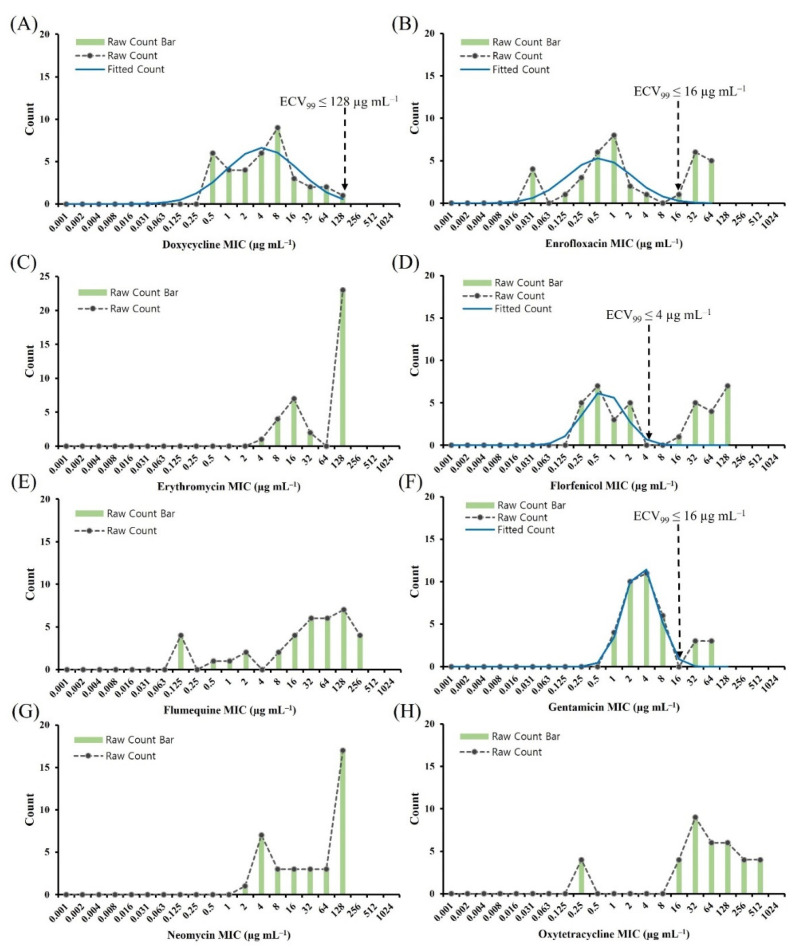
Distribution of MICs for *Aeromonas hydrophila*. MICs for *A. hydrophila* (*n* = 43) were determined using the broth microdilution method for (**A**) doxycycline, (**B**) enrofloxacin, (**C**) erythromycin, (**D**) florfenicol, (**E**) flumequine, (**F**) gentamicin, (**G**) neomycin, and (**H**) oxytetracycline. The blue raw-count bar and red dashed raw-count line indicate the observed number of isolates at each MIC, with the green fitted line of the MIC distribution modeled by ECOFFinder to include 99.0% of the WT isolates below the ECV. Vertical black dashed lines indicates the ECV_99_ determined in this study. Erythromycin, flumequine, neomycin, and oxytetracycline did not allow for ECV_99_ calculation. ECV, epidemiological cut-off value; MIC, minimum inhibitory concentration; WT, wild type.

Figure 2 shows the histogram of MICs for eight antimicrobial agents and the 99.0% ECV (ECV_99_), which was calculated using ECOFFinder software. The ECV_99_ value for doxycycline was 128 µg mL^−1^, indicating that no isolates could be considered NWT. The ECV_99_ value for enrofloxacin and gentamicin was 16 µg mL^−1^, which categorized 11 (25.6%) and six (14.0%) isolates as NWT, respectively. However, ECOFFinder failed to provide ECV_99_ values for four antimicrobial agents (erythromycin, flumequine, neomycin, and oxytetracycline) revealing the lack of a normal distribution; this complicated the interpretation of the MIC distributions.

**Figure 3:**
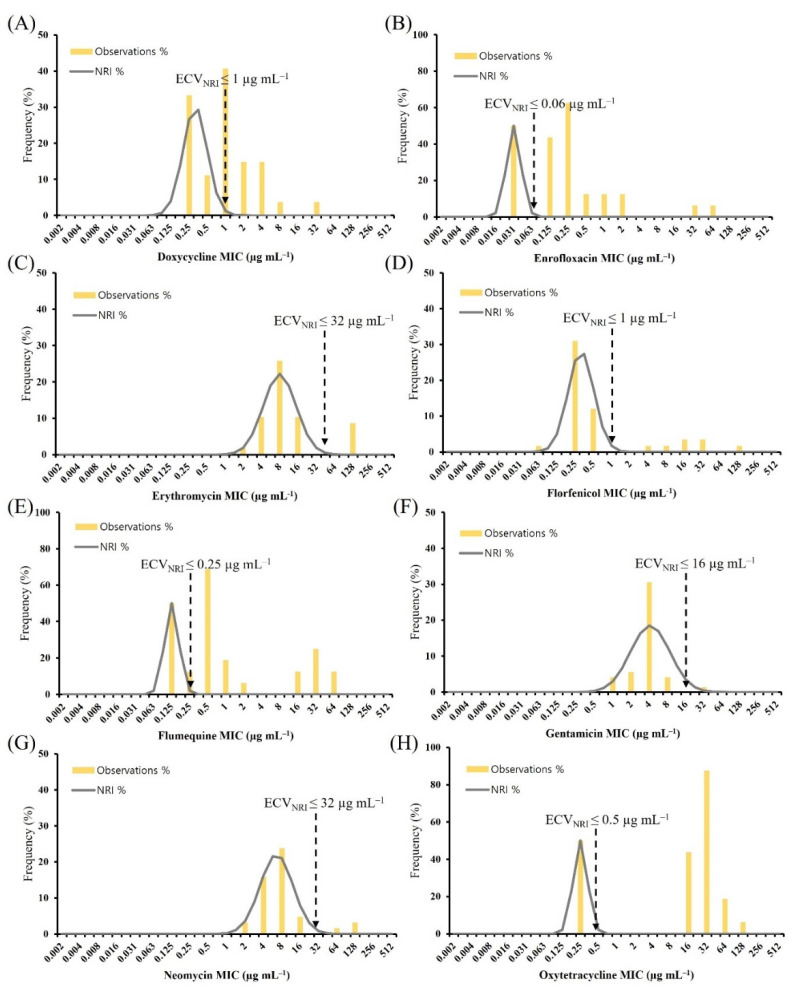
Distribution of MICs for *Aeromonas veronii*. MICs for *A. veronii* (*n* = 33) were determined using the broth microdilution method for (**A**) doxycycline, (**B**) enrofloxacin, (**C**) erythromycin, (**D**) florfenicol, (**E**) flumequine, (**F**) gentamicin, (**G**) neomycin, and (**H**) oxytetracycline. Gray lines indicate the NRI-derived normal distribution of WT isolates. Yellow vertical lines indicate the ECVs calculated from the data. Vertical black dashed lines indicate the ECV_NRI_ determined in this study. The standard deviations for eight antimicrobials were below 1.2 log_2_. ECV, epidemiological cut-off value; MIC, minimum inhibitory concentration; NRI, normalized resistance interpretation; WT, wild type.

Figure 3 shows the histogram of MIC for eight antimicrobial agents against *A. veronii* using the NRI method. The ECV_NRI_ values for doxycycline and enrofloxacin were 1 µg mL^−1^ and 0.06 µg mL^−1^, which categorized 10 (30.3%) and 25 (75.8%) isolates as NWT, respectively.

**Figure 4:**
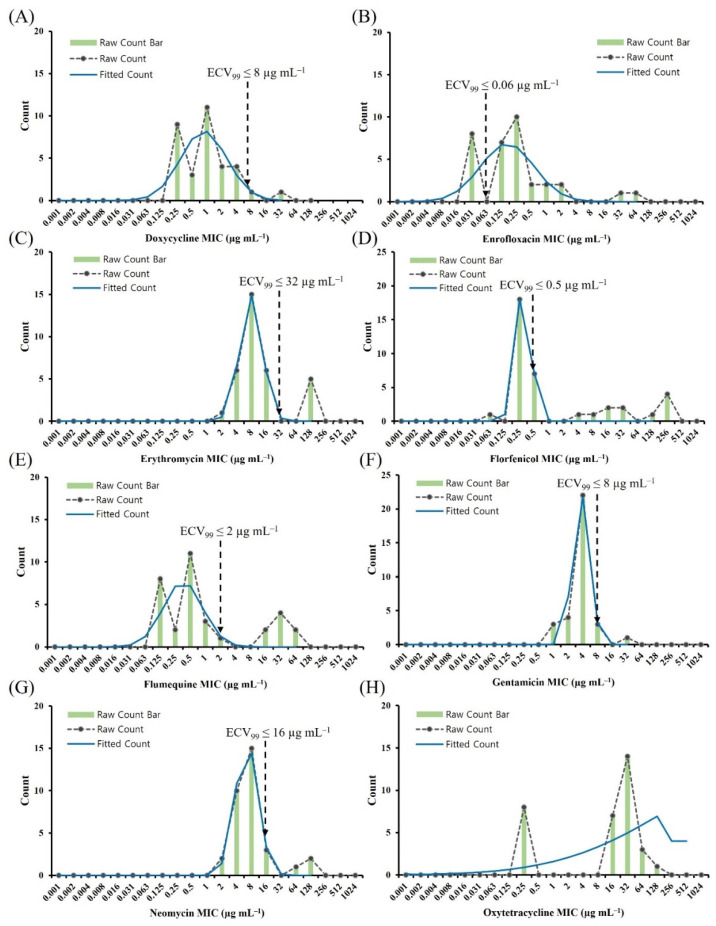
Distribution of MICs for *Aeromonas veronii*. MICs for *A. veronii* (*n* = 33) were determined using the broth microdilution method for (**A**) doxycycline, (**B**) enrofloxacin, (**C**) erythromycin, (**D**) florfenicol, (**E**) flumequine, (**F**) gentamicin, (**G**) neomycin, and (**H**) oxytetracycline. The blue raw- count bar and red dashed raw-count line depict the observed number of isolates at each MIC, with the green fitted line of the MIC distribution modeled by ECOFFinder to include 99.0% of the WT isolates below the ECV. Vertical black dashed lines indicate the ECV_99_ determined in this study. Oxytetracycline did not allow for ECV_99_ calculation. ECV, epidemiological cut-off value; MIC, minimum inhibitory concentration; WT, wild type.

Figure 4 shows the histogram of MICs for eight antimicrobial agents and the ECV_99_, The ECV_99_ values for florfenicol and flumequine were 0.5 µg mL^−1^ and 2 µg mL^−1^, which categorized seven (21.2%) and eight (24.2%) isolates as NWT, respectively. The ECV_99_ values for gentamicin and neomycin were 8 µg mL^−1^ and 16 µg mL^−1^, respectively.

### 2.3. Comparison of the ECV_CLSI_, ECV_NRI_, and ECV_99_

We compared the ECVs of eight antimicrobial agents for *A. hydrophila* and *A. veronii* isolates using the CLSI, NRI, and ECOFFinder methods. There is no breakpoint for the two *Aeromonas* spp. isolates; however, recently, the CLSI provided six ECVs for *A. hydrophila* [18]. The ECV_CLSI_ and ECV_NRI_ for erythromycin against *A. hydrophila*, was 64 µg mL^−1^ (Table 3). Additionally, the ECV_NRI_ and ECV_99_ for gentamicin was 16 µg mL^−1^, which was two-fold higher than ECV_CLSI_. The ECV_99_ for enrofloxacin was 16 µg mL^−1^, which was more than nine dilution steps from the ECV_CLSI_. Among the ECVs for the eight antimicrobials, the ECV for florfenicol was optimal, showing the least 1-fold dilution between ECV_CLSI_ and ECV_NRI_ or ECV_99_. We calculated values for flumequine and neomycin using only the NRI method. The CLSI has not provided the breakpoint or ECVs for *A. veronii*. The ECV_NRI_ and ECV_99_ values for enrofloxacin (0.06 µg mL^−1^) and erythromycin (32 µg mL^−1^) were the same (Table 4), whereas the ECV_NRI_ values for florfenicol, gentamicin, and neomycin were one-fold higher than the ECV_99_ values. Oxytetracycline was evaluated using only the NRI method with 0.5 µg mL^−1^ as the ECV_NRI_ value.

### 2.4. Presumptive Multidrug-Resistant (pMDR) Aeromonas spp. Isolates

A total of 18.6% (*n* = 8) of the isolates presented a pMDR phenotype, suggesting that multiple antimicrobial resistance is a common phenomenon in *A. hydrophila* (Table 5). All isolates from *Anguilla japonica*, *Silurus asotus*, *Salmo salar*, and *Misgurnus mizolepis* were resistant to three or more classes of antimicrobials. One isolate was resistant to seven antimicrobial agents, and five isolates were resistant to six agents. Additionally, 24.2% (*n* = 8) of *A. veronii* isolates presented the pMDR phenotype, and were highly resistant to enrofloxacin, florfenicol, and oxytetracycline. None of the isolates were resistant to all the eight antimicrobial agents.

### 2.5. Distribution of Antimicrobial Resistance Genes (ARGs)

**Figure 5:**
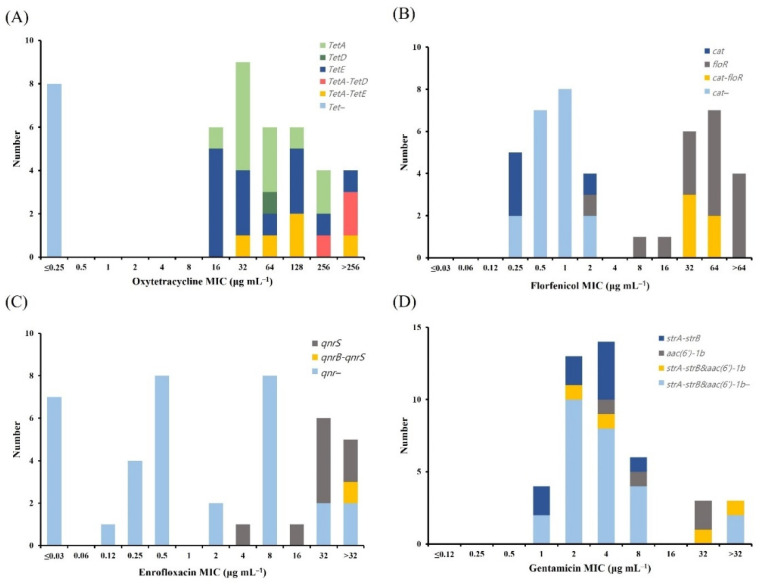
Distribution of ARGs among 43 *Aeromonas hydrophila* isolates. (**A**) Tetracycline-resistant genes (*tetA*, *tetB*, *tetD*, and *tetE*), (**B**) florfenicol-resistant genes (*cat* and *floR*), (**C**) quinolone-resistant genes (*qnrA*, *qnrB*, and *qnrS*), and (**D**) aminoglycoside-resistant genes (*strA-strB* and *aac(6′)-1b*). ARG, antimicrobial resistance gene.

**Figure 6:**
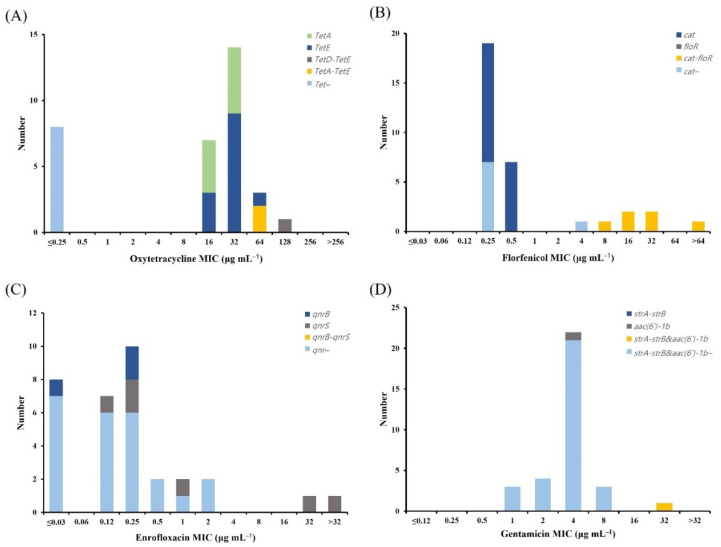
Distribution of ARGs among 33 *Aeromonas veronii* isolates. (**A**) Tetracycline-resistant genes (*tetA*, *tetB*, *tetD*, and *tetE*), (**B**) florfenicol-resistant genes (*cat* and *floR*), (**C**) quinolone-resistant genes (*qnrA*, *qnrB*, and *qnrS*), and (**D**) aminoglycoside-resistant genes (*strA-strB* and *aac(6′)-1b*). ARG, antimicrobial resistance gene.

We analyzed four *tet* genes (*tetA*, *tetB*, *tetD*, and *tetE*) encoding proteins involved in tetracycline efflux (Figure 5). In *A. hydrophila*, all the *tet*-positive isolates (35 isolates) were oxytetracycline NWT at ECV_CLSI_ (Figure 5A). The most common *tet* gene was *tetE*, which was found in 14 (40%) NWT isolates, followed by *tetA*, which was found in 12 (34.3%) NWT isolates. Some of the isolates carried more than one *tet* gene, with *tetA*–*tetD* (three isolates) and *tetA*–*tetE* (five isolates) related to the oxytetracycline MICs ranging from 32 µg mL^−1^ to 256 µg mL^−1^ and demonstrating high resistance to oxytetracycline. The *tetB* gene was not detected in any of the strains. We analyzed the four *tet* genes in *A. veronii* (Figure 6). In *A. veronii*, all the *tet*-positive isolates (25 isolates) were oxytetracycline NWT at ECV_NRI_ (Figure 6A), and the most common *tet* gene was *tetE*, which was found in 13 (52%) of the NWT isolates. Additionally, *A. veronii* isolates with MICs of 64 µg mL^−1^ (two strains) and 128 µg mL^−1^ (one strain) carried two *tet* genes, (*tetA*–*tetE* and *tetD*–*tetE*, respectively). The *tetB* gene was not detected in any of the strains.

Florfenicol NWT in *A. hydrophila* and *A. veronii* isolates was examined to determine the presence of the resistance genes for chloramphenicol acetyltransferase (*cat*) and florfenicol resistance (*floR*). In *A. hydrophila*, 79.2% (19/24) of the ARG-positive isolates were florfenicol NWT at ECV_99_ (Figure 5B), with *cat* and *floR* detected in 0% (0/19) and 73.7% (14/19) of the NWT isolates, respectively. Moreover, five isolates with MICs of 32 µg mL^−1^ and 64 µg mL^−1^ carried both the resistance genes (*cat*–*floR*). We detected no resistance genes in 19 isolates among all the strains. In *A. veronii*, 24.0% (6/25) of the florfenicol NWT at ECV_99_ were ARG-positive isolates (Figure 6B); however, 73.1% (19/26) of florfenicol WT carried the *cat* gene. Furthermore, six *A. veronii* isolates with MICs ranging from 8 µg mL^−1^ to 32 µg mL^−1^ and >64 µg mL^−1^ carried two resistant genes (*cat*–*floR*).

We tested *A. hydrophila* and *A. veronii* enrofloxacin NWT isolates for the three resistance genes, *qnrA*, *qnrB*, and *qnrS*. In *A. hydrophila*, 77.8% (7/9) of the ARG-positive isolates were enrofloxacin NWT at ECV_99_ (Figure 5C), with *qnrS* detected in 54.5% (6/11) of the NWT isolates; only one at MIC >32 µg mL^−1^ harbored more than one type of *qnr* gene. In *A. veronii*, 88.9% (8/9) of the ARG-positive isolates were enrofloxacin NWT at ECV_99_ (Figure 6C), with *qnrB* and *qnrS* detected in 8% (2/25) and 24% (6/25) of the NWT isolates, respectively. Furthermore, we did not detect the *qnrA* gene in any of the *A. hydrophila* or *A. veronii* strains.

We tested *A. hydrophila* and *A. veronii* gentamicin NWT isolates for the two resistance genes *strA-strB* and *aac(6′)-1b*. In *A. hydrophila*, 23.5% (4/17) of the ARG-positive isolates were gentamicin NWT at ECV_99_ (Figure 5D); however, 24.3% of the gentamicin WT 37 isolates carried the *strA-strB* gene. Among the NWT isolates, 66.7% harbored more than one resistance gene [e.g., *strA-strB*–*aac(6′)-1b*]. In *A. veronii*, there were no ARGs in the 31 isolates from the different strains (Figure 6D); however, one isolate with an MIC of 32 µg mL^−1^ harbored two resistant genes [*strA-strB*–*aac(6′)-1b*]. Table 6 summarizes the ARG distributions in the *A. hydrophila* and *A. veronii* isolates.

### 2.6. Quality Control (QC)

Eight antimicrobial agents of QC MICs for *Escherichia coli* ATCC 25922, *Aeromonas salmonicida* subsp. *salmonicida* ATCC 33658, and *Enterococcus faecalis* ATCC 29212 were within the acceptable range (94.3 to 100%) for the standard broth-microdilution method, as stipulated by the CLSI documents, M45, M7, and VET04 [18,24,25]. The results for doxycycline and neomycin against *A. salmonicida* ATCC 33658 were excluded from the QC, because of the lack of an established acceptable range in CLSI document VET04. Table S1 shows the MICs for the QC strains.

## 3. Discussion

The development of multiple antibiotic resistance strains of *A. hydrophila* and *A. veronii* in recent years is a serious public health concern, because of the possibility of their transmission from infected fish or water sources to humans and the subsequent infections [26]. In this study, we established eight ECVs for 43 *A. hydrophila* and 33 *A. veronii* isolates from aquatic animals and evaluated their ARG distributions. Some ECV_CLSI_ values were suggested for *A. hydrophila* [18]. The lack of clinical breakpoints or guidelines to interpret ECVs for *A. veronii* prompted the use of two methods for determining ECVs and interpreting the antimicrobial susceptibility of *A. hydrophila* and *A. veronii*.

Three antimicrobials (doxycycline, enrofloxacin, and oxytetracycline) exhibited bimodal MIC distributions, which revealed two clearly distinct populations of *A. hydrophila* and *A. veronii*. Based on these distributions, the calculated MIC_50_ (4 µg mL^−1^) for gentamicin against *A. hydrophila* and *A. veronii* was higher than 1 µg mL^−1^. This is in line with that reported for 138 *Aeromonas* spp. isolates recovered from European rivers [27]. The MIC_50_ and MIC_90_ values for oxytetracycline were 34.97 µg mL^−1^ and 149.26 µg mL^−1^, respectively, for 64 pathogenic *Aeromonas* strains isolated from ornamental fish [28]. Similarly, the MIC_50_ values were ≤2 µg mL^−1^ for florfenicol, 8 µg mL^−1^ for oxytetracycline, and 0.5 µg mL^−1^ for ciprofloxacin for 72 aeromonads isolated from koi carp [29]. These findings suggested that the isolates obtained 10 years ago were more susceptible to these drugs.

Tetracycline classes, including oxytetracycline and doxycycline, are broad-spectrum agents extensively used to treat bacterial infections and prevent infections in aquaculture. However, oxytetracycline is poorly absorbed in the fish gut; therefore, it must be administered at high doses [30]. This study showed that 89.2% of *A. hydrophila* could be categorized as NWT upon applying an oxytetracycline ECV_CLSI_ of 0.25 µg mL^−1^; 75.8% of *A. veronii* were determined as NWT upon applying an oxytetracycline ECV_NRI_ of 0.5 µg mL^−1^. This confirmed the high resistance rate in *Aeromonas* spp. However, 33 *Aeromonas* isolates (14.2%) recovered from 16 rivers were considered NWT for tetracycline (23), and 39 *Aeromonas* isolates (40.6%) from different fish species with reduced susceptibility to tetracycline were classified as NWT [23]. Additionally, *A. hydrophila* isolates from tilapia, carp, and channel catfish were more susceptible to doxycycline than to oxytetracycline [31]. *Aeromonas* spp. easily develop single or multiple antibiotic resistance phenotypes and are generally resistant to tetracyclines, quinolones, and β-lactams [5,32]. Moreover, tetracycline-resistant *Aeromonas* isolates are observed in wastewater discharge, lakes, and carp ponds [32,33,34,35]. In this study, we found that 62.8% of *A. hydrophila* isolates and 75.8% of *A. veronii* NWT isolates harbored *tetA*, *tetD*, *tetE*, or more than one *tet* gene, indicating that the WT isolates did not possess any *tet* genes. *Aeromonas* spp. isolates predominantly carried *tetE*, followed by *tetA*. However, 37% of *A. veronii* isolates recovered from channel catfish carried *tetE*, and 3.8% of isolates carried *tetA* [36]. Furthermore, *A. hydrophila* isolates showing oxytetracycline MICs ranging from 32–256 µg mL^−1^ harbored more than one *tet* gene (*tetA*-*tetE* and *tetA*-*tetD*), indicating that the degree of oxytetracycline resistance was associated with the number and type of *tet* genes present. *E. coli* isolates harboring *tetA* and *tetB* or *tetA* and *tetC* exhibited high MICs for tetracycline (256 µg mL^−1^) or oxytetracycline (512 µg mL^−1^) [37]. The ECV_CLSI_ for *A. hydrophila* and ECV_NRI_ for *A. veronii* might account for the correlations between the NWT isolates and the distribution of resistance genes.

In Korea, florfenicol is approved for use against bacterial diseases in *Oncorhynchus mykiss*, *A. japonica*, and *Seriola quinqueradiata* [38]. The ECV_NRI_ for florfenicol is 1 µg mL^−1^ for *A. hydrophila* (55.8%) and *A. veronii* (21.2%), which were categorized as NWT with reduced susceptibility. However, 2.1% isolates of *Aeromonas* spp. are NWT considering the ECV_NRI_ (2 µg mL^−1^) [21], and 25.5% are NWT considering the ECV_NRI_ (4 µg mL^−1^) [23]. The high frequency of NWT isolates from Korea could be associated with the excessive use of antimicrobial agents in aquaculture; the recorded florfenicol sales was approximately six tons in 2019 [39]. Additionally, we detected *cat* and *floR* in *A. hydrophila* and *A. veronii* NWT isolates; both genes are associated with high MICs. A total of 7.5% *A. veronii* isolates harbored *floR*, which conferred resistance to florfenicol [36]. A resistance cassette, carrying the *floR* gene in *A. salmonicida* enables mobilization [40]. The first *floR*-containing plasmid was discovered in *Aeromonas bestiarum* [41]. Interestingly, the presence of *cat* was related to a low MIC for florfenicol (0.25 or 0.5 µg mL^−1^). These results indicated a higher correlation between the presence of *floR* and NWT categorization, compared to that with the presence of *cat*.

Enrofloxacin is a member of the fluoroquinolone family of antibiotics and exhibits strong bactericidal activity against aerobic and facultative anaerobic bacteria [42]. For *A. hydrophila*, the ECV_CLSI_ of 0.03 µg mL^−1^ was lower than the ECV_99_ of 16 µg mL^−1^, indicating that lowering the ECV would increase the likelihood of identifying resistance genes or mutants while increasing the risk of misclassifying the number of WT isolates. Based on our findings, an ECV_CLSI_ of 0.03 µg mL^−1^ would misclassify 58.1% of NWT (25 isolates), compared to an ECV_99_ of 16 µg mL^−1^. We mostly detected *qnrS* in *A. hydrophila* and *A. veronii* NWT isolates; therefore, ECVs should be established in detail based on the ARG distributions. *qnrS* was the most prevalent, with its presence in 68% of aeromonad isolates that demonstrated high levels of resistance to nalidixic acid and ciprofloxacin; no amplicon was detected for *qnrA* [43]. The detection of the factors enabling plasmid-mediated quinolone resistance indicated that the complex *Aeromonas* mobilome increases the possibility of horizontal gene transfer, including that of *qnrS* and *qnrB*.

Erythromycin is not approved for use in the USA; however, *Aeromonas* strains highly resistant to erythromycin have been isolated from foreign countries [44]. Additionally, *Aeromonas* spp. are resistant to penicillin, cephalosporins, vancomycin, and erythromycin [45,46]. In this study, 53.5% and 15.2% of *A. hydrophila* and *A. veronii*, respectively, were categorized as NWT upon application of the erythromycin ECV_NRI_. Similarly, 50% and 53% of aeromonads isolated from lakes and chickens, respectively, showed resistance to erythromycin [47,48]. Furthermore, harboring macrolide *MacB ABC* transporter genes confers erythromycin resistance; the *MacA* gene regulates the drug-binding and ATPase activity of *MacB* [49]. We did not investigate the distribution of macrolide resistance genes; further studies are required to elucidate the cause underlying the acquisition of erythromycin resistance, owing to the high prevalence of erythromycin NWT *Aeromonas* spp. isolates.

The results showed that 3% of *A. veronii* was classified as NWT upon application of the gentamicin ECV_NRI_ and ECV_99_. Consistent with these findings, 2% of *Aeromonas* spp. exhibited gentamicin resistance; however, no *A. veronii* isolate was resistant to gentamicin [50]. We did not detect any aminoglycoside- resistance genes among the 31 A. veronii isolates (94%). However, in an earlier report, all *Aeromonas* spp. isolates recovered from marketed cockles harbored *aac(6′)-1b*, with *strA-strB* found in 41% of the isolates [43]. The recommended first-line therapeutic options for *Aeromonas* infections are aminoglycosides and fluoroquinolones. We identified gentamicin as an aquatic medicine that can be inoculated orally to prevent *Aeromonas* infection. Its appropriate use could potentially prevent the emergence of new resistant strains.

The resistance phenotypes varied among isolates. The pMDR of *A. hydrophila*, which was resistant to three or more classes of antimicrobials, was 18.6%; this was lower than that observed in a previous study conducted on tilapia where 64% of isolates were resistant to six to eight drugs [31] and that in 95 motile pMDR aeromonads isolated from freshwater [46]. Additionally, multi-antibiotic resistant *Aeromonas* spp. isolates harbored a tripartite AheABC efflux pump, and the use of phenylalanine–arginine–β–naphthylamide contributed to intrinsic resistance [51]. Among the *Aeromonas* spp. isolates identified as pMDR, the most common resistance was against oxytetracycline (100%). Oxytetracycline is among the most commonly used antibiotics in humans and animals, and these results are consistent with those of a previous study [52]. The distribution of strains resistant to oxytetracycline has increased with the global use of antibiotics; the emergence of pMDR strains complicates the selection of available therapeutics.

This study provides eight putative ECVs for classifying WT and NWT isolates; however, the findings should not be used as *Aeromonas*-pathogen-treatment guidelines. These ECVs were derived from one laboratory; therefore, it is essential to evaluate different sources and a large number of isolates for reliably establishing ECVs for each *Aeromonas* strain [53]. The results from this study can be used as a foundation to establish clinical breakpoints for each *Aeromonas* strain. Additionally, it is necessary to study the NWT bacterial transcriptome and the mechanism of antibiotic resistance transmission between humans and fish to determine the cause of resistance acquisition.

## 4. Materials and Methods

### 4.1. Collection and Isolation of Aeromonas spp.

**Figure 7:**
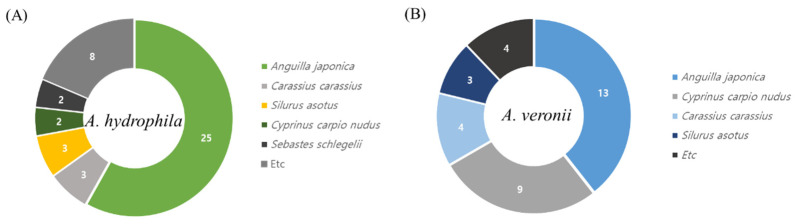
Isolated strains used in this study. These included (**A**) 43 *Aeromonas hydrophila* and (**B**) 33 *Aeromonas veronii* strains isolated from various aquatic animals from Korea.

*Aeromonas* spp. isolates were collected between 2008 and 2020 from eight Korean provinces (Chungbuk, Chungnam, Gyeongbuk, Gyeongnam, Gyeonggi, Jeonbuk, Jeonnam, and Gangwon), with 43 *A. hydrophila* isolates recovered from *A. japonica* (*n* = 25), *Carassius carassius* (*n* = 3), *S. asotus* (*n* = 3), *Cyprinus carpio nudus* (*n* = 2), *Sebastes schlegelii* (*n* = 2), and others (*n* = 8); and 33 *A. veronii* isolates recovered from *A. japonica* (*n* = 13), *C. carpio nudus* (*n* = 9), *C. carassius* (*n* = 4), *S. asotus* (*n* = 3), and others (*n* = 4) (Figure 7). The bacterial strains are listed in Tables S2 and S3. The fish species were sampled from among seemingly healthy, clinical–subclinical, and moribund fish that differed by the year and region of collection. Samples were taken from the lesions, kidneys, and spleens of fish. All experiments were performed in accordance with Directive 2010/63/EU of the European Parliament and the Council (22 September 2010) on the protection of animals used for scientific purposes. *Aeromonas* spp. isolates were grown on *Aeromonas agar* (MB cells, Los Angeles, CA, USA), incubated at 37 °C for 24 h. Presumptive aeromonad colonies showing typical dark-green opaque color with a dark center were chosen and subjected to molecular identification. Genomic DNA was extracted from a single colony using a QIAmp DNA blood mini kit (Qiagen, Milan, Italy), according to the manufacturer instructions. DNA concentration and purity were quantified using a Nano Drop R 2000 spectrophotometer (Thermo Fisher Scientific, Waltham, MA, USA) DNA was stored at −80 °C until use. *Aeromonas* spp. isolates were stored at −80 °C in tryptic soy broth (Merck, Kenilworth, NJ, USA) supplemented with 20% glycerol until further use.

### 4.2. Molecular Identification

Bacterial identities were confirmed using PCR with two different primer sets for amplification and sequencing of 16S rRNA and *gyrB*. The 16s rRNA gene (1361 bp) was amplified and sequenced using specific primers (27F: 5′-AGA GTT TGA TCM TGG CTC AG-3′ and 1387R: 5′-GGG CGG WGT GTA CAA GGC-3′). *gyrB* (904 bp) was used as the housekeeping gene to further identify species (*gyrB* 3F: 5′-TCC GGC GGT CTG CAC GGC GT-3′ and *gyrB* 14R: 5′-TTG TTC GGG TTG TAC TCG TC-3′) [54]. The PCR reaction mix at 50 µL contained 5 µL of 10× Ex Taq buffer, 4 µL dNTP mixture (2.5 mM each), 10 pmol of each primer, 0.25 µL Ex Taq DNA polymerase (Takara, Shiga, Japan), 10 ng DNA template, and sterile purified water. The reaction conditions were as follows: initial denaturation at 95 °C for 3 min, followed by 30 cycles of denaturation at 98 °C for 10 s, 55 °C for 30 s, and extension at 72 °C for 30 s, with a final extension at 72 °C for 7 min. The PCR products were confirmed through sequence analyses (Bionics, Seoul, Korea); the strains were verified based on the reference sequences accessed from GenBank (https://www.ncbi.nlm.nih.gov/genbank/) (accessed on: 5 May 2021).

### 4.3. Antimicrobial Susceptibility Test

Antimicrobial susceptibility tests were performed according to the broth microdilution method described in the CLSI guidelines VET04 [17,18]. The antimicrobial agents for *Aeromonas* spp. isolates are licensed and commonly used for aquatic animals in Korea [38]. The MICs of 43 *A. hydrophila* and 33 *A. veronii* isolates were tested using Sensititre CAMPY2 and KRAQ1 plates (Trek Diagnostics System, Cleveland, OH, USA). MICs for erythromycin (0.03–64 mg L^−1^), florfenicol (0.03–64 mg L^−1^), and gentamicin (0.12–32 mg L^−1^) were tested using CAMPY2; and those for doxycycline (0.25–64 mg L^−1^), enrofloxacin (0.03–32 mg L^−1^), flumequine (0.12–128 mg L^−1^), neomycin (0.5–64 mg L^−1^), and oxytetracycline (0.25–256 mg L^−1^) were tested using KRAQ1. Isolates were cultured on tryptic soy agar for 24 h at 28 °C, after which a suspension was prepared in sterile saline solution, adjusted to 0.5 McFarland standard, and diluted to reach a final inoculum concentration of 5 × 10^5^ CFU/mL using a Nephelometer^®^ (V3011, Thermo Scientific, Roskilde, Denmark)) to standardize inoculum density/turbidity. Microplates were incubated at 28 °C for 24 h for *A. hydrophila* and *A. veronii*. MICs were defined as the lowest drug concentrations that inhibited growth, compared to that in the drug-free growth control. *E. coli* ATCC 25922, *A. salmonicida* subsp. *salmonicida* ATCC 33658, and *E. faecalis* ATCC 29212 were included in the susceptibility test as QC strains. Recently, additional MICs of ECVs were made available for *A. hydrophila* in the updated CLSI guidelines [18]. We compared the *A. hydrophila* and *A. veronii* isolates among WT and NWT populations, according to the CLSI guidelines and the provisional ECVs proposed in this study.

### 4.4. Determination of Provisional ECVs

ECVs were calculated using two methods: NRI [55] and ECOFFinder [56]. The NRI method is a fully automatic and freely available Excel spreadsheet calculator (last updated in 2019; http://www.bioscand.se/nri) (accessed on: 3 May 2021). The ECOFFinder method (v.2.1; last updated in 2020) is available from the EUCAST website (https://www.eucast.org/mic_distributions_and_ecoffs) (accessed on: 3 May 2021). In this study, ECV determination was based on the distribution of antimicrobial MICs for each drug against *A. hydrophila* and *A. veronii*. ECV allows isolates to be categorized as WT at ≤x mg L^−1^ and NWT as >x mg L^−1^. A 99.0% cut-off was applied, which means that approximately 99.0% of the WT MIC distribution was less than the identified ECV. pMDR was defined as resistance to more than three antimicrobial agents, classes, or subclasses of antimicrobial categories [57]. The number of pMDR *Aeromonas* was determined for eight antimicrobial agents (doxycycline, enrofloxacin, erythromycin, florfenicol, flumequine, gentamicin, neomycin, and oxytetracycline) in the clinical samples.

### 4.5. Terminology

When referring to the categorization of isolates based on their susceptibility, we followed the recommendations, which suggested that when isolates are categorized by applying ECVs, the terms “sensitive” and “resistant” should not be used [58]. WT is defined, for a fully susceptible species, as the absence of acquired- and mutational-resistance mechanisms to the drug, and NWT is defined as the reduced susceptibility to the presence of an acquired- or mutational- resistance mechanism to the drug. However, when referring to studies that used the term “resistant”, we did not change their terminology. The CLSI uses the abbreviation “ECV” for epidemiological cut-off values, whereas EUCAST uses the ECOFF. This study used “ECV” to prevent confusion when comparing the ECOFF values using the two analytical methods.

### 4.6. Analysis of ARGs

We tested 43 *A. hydrophila* and 33 *A. veronii* isolates for the presence of ARGs, including *tetA*, *tetB*, *tetD*, and *tetE* for tetracycline; *cat* and *floR* for phenicol; *qnr*-type pentapeptide proteins encoded by *qnrA*, *qnrB*, and *qnrS* for quinolone; and *strA-strB* and *aac(6′)-1b* for aminoglycosides (Table 7). The primers used to detect these genes were selected from previous studies. The PCR cycling conditions were as follows: 94 °C for 5 min, followed by 35 cycles of 95 °C for 30 s, annealing for 30 s at different temperatures, 72 °C for 30 s, and 72 °C for 5 min. The PCR products were separated using electrophoresis on a 1% agarose gel and purified for sequencing. Sequence identities were confirmed using the sequence information in the NCBI database (on https://www.ncbi.nlm.nih.gov/) (accessed date: 22 June 2021).

## 5. Conclusions

This is the first study to establish ECV_NRI_ and ECV_99_ values for eight antimicrobials against 43 *A. hydrophila* and 33 *A. veronii* isolates recovered from aquatic animals in Korea and to detect ARGs in *Aeromonas* strains. A total of 89.2% *A. hydrophila* isolates and 75.8% *A. veronii* isolates were classified as NWT against oxytetracycline; they harbored *tet genes*; *Aeromonas* spp. isolates predominantly carried *tetE*, followed by *tetA*. Additionally, the distribution of *floR* and *qnrS* was prevalent in NWT isolates, whereas no *aac(6′)-1b* or *strA-strB* was detected in the 31 *A. veronii* isolates. The emergence of antibiotic-resistant strains of *Aeromonas* spp. reduces the choice of currently available therapeutic agents and it could lead to prolonged *Aeromonas* infections. Therefore, these results can potentially help aquaculture managers and researchers alleviate *Aeromonas* infections in aquaculture systems and raise awareness of the appropriate use of antimicrobials in aquaculture. Furthermore, these findings encourage the application of vaccination or herbal therapy, to reduce antibiotic resistance and public health problems.

## Figures and Tables

**Table 1:** MIC distribution of antimicrobial agents in 43 *Aeromonas hydrophila* isolates obtained from aquatic animals in Korea.

Antimicrobials	No. of Isolates with MIC ^a^ (µg mL^−1^)															MIC_50_	MIC_90_
	0.03	0.06	0.12	0.25	0.5	1	2	4	8	16	32	64	128	256	512		
Doxycycline				8	3	5	4	6	9	3	2	2	1			4	32
Enrofloxacin	7	0	1	4	8	8	2	1	0	1	6	5				1	32<
Erythromycin	0	0	0	0	0	0	0	1	4	8	4	3	23			64<	64<
Florfenicol	0	0	0	5	7	7	5	0	1	1	5	5	7			2	64<
Flumequine			7	0	1	2	2	0	2	5	7	6	7	4		32	128
Gentamicin			0	0	0	4	13	14	6	0	3	3				4	32
Neomycin					0	0	3	10	4	3	3	3	17			32	64<
Oxytetracycline				8	0	0	0	0	0	6	9	6	6	4	4	64	256

^a^ MIC, minimum inhibitory concentration. White fields represent the range of the dilutions tested.

**Table 2:** MIC distribution of antimicrobial agents in 33 *Aeromonas veronii* isolates obtained from aquatic animals in Korea.

Antimicrobials	No. of Isolates with MIC ^a^ (µg mL^−1^)															MIC_50_	MIC_90_
	0.03	0.06	0.12	0.25	0.5	1	2	4	8	16	32	64	128	256	512		
Doxycycline				9	3	11	4	4	1	0	1	0	0			1	4
Enrofloxacin	8	0	7	10	2	2	2	0	0	0	1	1				0.25	2
Erythromycin	0	0	0	00	0	0	1	6	15	6	0	0	5			8	64<
Florfenicol	0	1	0	18	7	0	0	1	1	2	2	0	1			0.25	16
Flumequine			8	2	11	3	1	0	0	2	4	2	0	0		0.5	16
Gentamicin			0	0	0	3	4	22	3	0	1	0				4	8
Neomycin					0	0	2	10	15	3	0	1	2			8	16
Oxytetracycline				8	0	0	0	0	0	7	14	3	1	0	0	32	64

^a^ MIC, minimum inhibitory concentration. White fields represent the range of the dilutions tested.

**Table 3:** Comparison of the ECVs of eight antimicrobial agents for *Aeromonas hydrophila* isolates based on the CLSI, NRI, and ECOFFinder methods.

Species	Antimicrobial	ECV_CLSI_ (µg mL^−1^)	WT(%)	NWT(%)	ECV_NRI_ (µg mL^−1^)	WT(%)	NWT(%)	ECV_99_ (µg mL^−1^)	WT(%)	NWT(%)
*A. hydrophila*	Doxycycline	ND	-	-	2	46.5	53.5	128	100.0	0.0
	Enrofloxacin	0.03	16.3	83.7	32 ^#^	88.4	11.6	16	74.4	25.6
	Erythromycin	64	46.5	53.5	64	46.5	53.5	ND	-	-
	Florfenicol	2	55.8	44.2	1	44.2	55.8	4	55.8	44.2
	Flumequine	ND	-	-	64 ^#^	74.4	25.6	ND	-	-
	Gentamicin	4	72.1	27.9	16	86.0	14.0	16	86.0	14.0
	Neomycin	ND	-	-	16	46.5	53.5	ND	-	-
	Oxytetracycline	0.25	18.6	62.8	ND	-	-	ND	-	-

^#^ Standard deviation >1.2 log_2_. CLSI, Clinical and Laboratory Standards Institute; ECV, epidemiological cut-off value; ND, not possible to determine the ECV; NWT, non-wild type; WT, wild type.

**Table 4:** Comparison of the ECVs of eight antimicrobial agents for *Aeromonas veronii* isolates based on the CLSI, NRI, and ECOFFinder methods.

Species	Antimicrobial	ECV_CLSI_ (µg mL^−1^)	WT(%)	NWT(%)	ECV_NRI_ (µg mL^−1^)	WT(%)	NWT(%)	ECV_99_ (µg mL^−1^)	WT(%)	NWT(%)
*A. veronii*	Doxycycline	ND	-	-	1	69.7	30.3	8	97.0	3.0
	Enrofloxacin	ND	-	-	0.06	24.2	75.8	0.06	24.2	75.8
	Erythromycin	ND	-	-	32	84.8	15.2	32	84.8	15.2
	Florfenicol	ND	-	-	1	78.8	21.2	0.5	78.8	21.2
	Flumequine	ND	-	-	0.25	30.3	69.7	2	75.8	24.2
	Gentamicin	ND	-	-	16	97.0	3.0	8	97.0	3.0
	Neomycin	ND	-	-	32	90.9	9.1	16	90.9	9.1
	Oxytetracycline	ND	-	-	0.5	24.2	75.8	ND	-	-

CLSI, Clinical and Laboratory Standards Institute; ECV, epidemiological cut-off value; ND, not possible to determine the ECV; NWT, non-wild type; WT, wild type.

**Table 5:** pMDR profiles of *Aeromonas hydrophila* and *Aeromonas veronii* isolates collected from aquatic animals.

Strain	Isolate No.	Host	Year	Phenotype
*A. hydrophila*	20FBAer0358	*Anguilla japonica*	2020	E, Er, F, Fl, G, N, O
	20FBAer0371	*Anguilla japonica*	2020	Er, F, Fl, G, N, O
	20FBAer0351	*Anguilla japonica*	2020	E, F, G, O
	19FBAHy0001	*Silurus asotus*	2019	E, Er, F, Fl, N, O
	18FBAHy0001	*Silurus asotus*	2018	E, Er, F, Fl, N, O
	18FBAhy0003	*Anguilla japonica*	2018	E, Er, F, Fl, N, O
	17FBAHy0013	*Salmo salar*	2017	E, Er, F, Fl, N, O
	17FBAHy0006	*Misgurnus mizolepis*	2017	F, G, N, O
*A. veronii*	20FBAer0306	*Anguilla japonica*	2020	E, F, G, N, O
	20FBAer0374	*Oncorhynchus mykiss*	2020	E, Er, N, O
	21FBAer0172	*Cyprinus carpio nudus*	2018	E, Er, F, Fl, N, O
	21FBAer0163	*Cyprinus carpio nudus*	2018	E, F, Fl, O
	21FBAer0164	*Carassius carassius*	2018	E, F, Fl, O
	21FBAer0171	*Cyprinus carpio nudus*	2018	E, F, O
	FP3978	*Cyprinus carpio nudus*	2010	D, E, Er, Fl, O
	FP3973	*Cyprinus carpio nudus*	2010	E, F, O

D, doxycycline; E, enrofloxacin; Er, erythromycin; F, florfenicol; Fl, flumequine; G, gentamicin; N, neomycin; O, oxytetracycline; pMDR, presumptive multidrug-resistant.

**Table 6:** ARG distribution in *Aeromonas hydrophila* and *Aeromonas veronii*.

	Tetracycline					Florfenicol			Quinolone				Aminoglycoside		
	*tetA*	*tetB*	*tetD*	*tetE*	Others *	*cat*	*floR*	Others *	*qnrA*	*qnrB*	*qnrS*	Others *	*strA-strB*	*aac(6′)-1b*	Others *
*A. hydrophila*	12	-	1	14	8	4	15	5	-	-	8	1	9	4	4
*A. veronii*	9	-	0	13	3	19	-	6	-	3	6	-	-	1	1

* Indicates the presence of >1 ARG: *tetA*–*tetD*, *tetA*–*tetE*, *tetD*–*tetE*; *cat*–*floR*; *qnrB*–*qnrS*; *strA-strB*–*aac(6’)-1b*.

**Table 7:** PCR primers to detect ARGs.

Class	Primer	Sequence (5′–3′)	AT * (°C)	Size(bp)	Reference
Tetracycline	*tetA*-F	GCG CTN TAT GCG TTG ATG CA	53	387	[59]
	*tetA*-R	ACA GCC CGT CAG GAA ATT			
	*tetB*-F	CTC AGT ATT CCA AGC CTT TG	58	400	[60]
	*tetB*-R	CTA AGC ACT TGT CTC CTG TT			
	*tetD*-F	GCG CTN TAT GCG TTG ATG CA	50	484	[59]
	*tetD*-R	CCA GAG GTT TAA GCA GTG T			
	*tetE*-F	GCG CTN TAT GCG TTG ATG CA	50	246	[59]
	*tetE*-R	ATG TGT CCT GGA TTC CT			
Phenicol	*cat*-F	AGC GCA ACG TCC TCT ATC AC	55	378	This study (PMU05929.1)
	*cat*-R	TGT CGT CGT CAA AGC GGT AG			
	*floR*-F	GCC CGC TAT GAT CCA ACT CA	55	289	This study (QEV84023.1)
	*floR-R*	AAG GCC GTA GAT GAC GAC AC			
Quinolone	*qnrA*-F	AGA GGA TTT CTC ACG CCA GG	56	580	[61]
	*qnrA*-R	TGC CAG GCA CAG ATC TTG AC			
	*qnrB*-F	GAT CGT GAA AGC CAG AAA GG	53	496	[61]
	*qnrB*-R	ACG ATG CCT GGT AGT TGT CC			
	*qnrS*-F	GCA AGT TCA TTG AAC AGG GT	56	428	[61]
	*qnrS*-R	TCT AAA CCG TCG AGT TCG GCG			
Aminoglycoside	*strA-strB*-F	TAT CTG CGA TTG GAC CCT CTG	55	538	[62]
	*strA-strB*-R	CAT TGC TCA TCA TTT GAT CGG CT			
	*aac(6′)-1b*-F	TTG CGA TGC TCT ATG AGT GGC TA	55	482	[63]
	*aac(6′)-1b*-R	CTC GAA TGC CTG GCG TGT TT			

* AT; annealing temperature.

## Data Availability

The data that support the findings of this study are available upon request from the corresponding author.

## Supplementary Materials

The following supporting information can be downloaded at: https://www.mdpi.com/article/10.3390/antibiotics11030343/s1, Table S1: CLSI-approved broth microdilution MIC QC ranges determined for eight antimicrobial agents against selected reference strains; Table S2: Isolate year, fish species, disease outbreak, isolation source, and geographical location of the 43 *A. hydrophila* strains; Table S3: Isolate year, fish species, disease outbreak, isolation source, and geographical location of the 33 *A. veronii* strains.
