# Supplementary material for: Establishment of Epidemiological Cut-Off Values and the Distribution of Resistance Genes in Aeromonas hydrophila and Aeromonas veronii Isolated from Aquatic Animals
Source: Antibiotics (Basel). 2022 Mar 5;11(3):343. doi: 10.3390/antibiotics11030343 (PMC8944483; doi:10.3390/antibiotics11030343)
Supplement: Supplementary file 1 [file antibiotics-11-00343-s001.zip › antibiotics-1575797-supplementary.pdf]

Table S1. CLSI-approved broth microdilution MIC QC ranges determined for eight antimicrobial agents against selected reference strains

Reference strain	Antimicrobial agent	MIC QC range (μg mL $^{-1}$)[a]	No. of doubling dilutions in range	% of values within range[b]
Escherichia coli	Doxycycline	0.5−2[c]	-	-
ATCC 25922	Enrofloxacin	0.004−0.015	3	100
	Erythromycin	ND	-	-
	Florfenicol	2−16	4	94.6
	Flumequine	0.06−0.5	4	100
	Gentamicin	0.12−0.5	3	100
	Neomycin	0.5−4[d]	4	97.1
	Oxytetracycline	0.25−1	3	100
Aeromonas salmonicida	Doxycycline	ND	-	-
subsp. *salmonicida*	Enrofloxacin	0.008−0.03	3	100
ATCC 33658	Erythromycin	4−16	3	100
	Florfenicol	0.25−1	3	100
	Flumequine	0.015−0.12	4	100
	Gentamicin	0.25−1	3	99.6
	Neomycin	ND	-	-
	Oxytetracycline	0.06−0.25	3	100
Enterococcus faecalis ATCC 29212	Erythromycin	1−4 [c]	3	100

[a] Acceptable QC ranges of MIC for references strain in CLSI VET04.

[b] Percentage of values in range determined from 80 replicates performed in this study.

[c] Acceptable QC ranges of MIC for references strain in CLSI M7 (M100) and M45.

[d] QC ranges of MIC for references s3train in Sensititre® Development Range.

*ND; not determined.

Table S2. Isolate year, fish species, disease outbreak, isolation source, and geographical location of the 43 *A. hydrophila* strains

No.	Isolate No.	Year	Fish species	Disease outbreak	Isolation source	Geographical location
1	20FBAer0010	2020	*Anguilla japonica*	Mar	Kidney	Jeonnam
2	20FBAer0160	2020	*Cyprinus carpio nudus*	Jun	Kidney	Gyeongnam
3	20FBAer0311	2020	*Sebastes schlegelii*	Oct	Kidney	Chungnam
4	20FBAer0312	2020	*Sebastes schlegelii*	Oct	Kidney	Chungnam
5	20FBAer0325	2020	*Oncorhynchus mykiss*	Feb	Kidney	Gangwon
6	20FBAer0351	2020	*Anguilla japonica*	Jun	Spleen	Gangwon
7	20FBAer0352	2020	*Anguilla japonica*	Jun	Spleen	Gangwon
8	20FBAer0353	2020	*Anguilla japonica*	Jun	Kidney	Gangwon
9	20FBAer0358	2020	*Anguilla japonica*	Jun	Kidney	Gangwon
10	20FBAer0369	2020	*Carassius carassius*	Jul	Spleen	Gangwon
11	20FBAer0371	2020	*Anguilla japonica*	Jul	Kidney	Gangwon
12	19FBAHy0001	2019	*Silurus asotus*	May	Kidney	Chungnam
13	19FBAHy0002	2019	*Silurus asotus*	May	Spleen	Gyeongbuk
14	19FBAHy0003	2019	*Anguilla japonica*	May	Kidney	Gyeonggi
15	19FBAHy0005	2019	*Carassius carassius*	Apr	Kidney	Gyeonggi
16	19FBAHy0006	2019	*Anguilla japonica*	Jul	Kidney	Chungnam
17	19FBAHy0007	2019	*Carassius carassius*	Jul	Spleen	Gyeonggi
18	19FBAHy0004	2018	*Anguilla japonica*	Jul	Spleen	Gangwon
19	18FBAHy0001	2018	*Silurus asotus*	May	Spleen	Gangwon
20	18FBAHy0002	2018	*Pelteobagrus fulvidraco*	May	Kidney	Gyeongbuk
21	18FBAhy0003	2018	*Anguilla japonica*	Sep	Spleen	Jeonnam
22	21FBAer0168	2018	*Cyprinus carpio*	May	Spleen	Gyeongnam
23	21FBAer0173	2018	*Cyprinus carpio*	July	Kidney	Gyeongnam
24	21FBAer0184	2018	*Anguilla japonica*	Sep	Kidney	Gyeongnam
25	21FBAer0188	2018	*Anguilla japonica*	Sep	Spleen	Gyeongnam
26	21FBAer0191	2018	*Anguilla japonica*	Sep	Kidney	Gyeongnam

27	21FBAer0192	2018	*Anguilla japonica*	Sep	Kidney	Gyeongnam
28	17FBAHy0001	2017	*Anguilla japonica*	Jun	Kidney	Jeonnam
29	17FBAHy0002	2017	*Anguilla japonica*	Jun	Kidney	Jeonnam
30	17FBAHy0003	2017	*Anguilla japonica*	Jun	Kidney	Jeonbuk
31	17FBAHy0006	2017	*Misgurnus mizolepis*	May	Spleen	Jeonbuk
32	17FBAHy0007	2017	*Cyprinus carpio*	May	Kidney	Gyeongnam
33	17FBAHy0008	2017	*Siniperca scherzeri*	May	Spleen	Gyeongnam
34	17FBAHy0009	2017	*Anguilla japonica*	Jul	Kidney	Jeonnam
35	17FBAHy0011	2017	*Anguilla japonica*	Aug	Spleen	Jeonnam
36	17FBAHy0012	2017	*Anguilla japonica*	Sep	Kidney	Jeonnam
37	17FBASa0016	2017	*Salmo salar*	Oct	Kidney	Jeonnam
38	FPa4559	2013	*Anguilla japonica*	May	Spleen	Jeonnam
39	FPa4560	2013	*Anguilla japonica*	May	Kidney	Jeonnam
40	FP3969	2010	*Cyprinus carpio nudus*	Jul	Kidney	Jeonnam
41	FP3142	2010	*Anguilla japonica*	Jul	Spleen	Jeonnam
42	FP7045	2010	*Anguilla japonica*	Jul	Kidney	Jeonnam
43	FP2363	2008	*Anguilla japonica*	Jul	Kidney	Jeonnam

Table S3. Isolate year, fish species, disease outbreak, isolation source, and geographical location of the 33 *A. veronii* strains

No.	Isolate No.	Year	Fish species	Disease outbreak	Isolation source	Geographical location
1	20FBAer0306	2020	*Anguilla japonica*	Sep	Kidney	Gyeongnam
2	20FBAer0374	2020	*Oncorhynchus mykiss*	Aug	Kidney	Gangwon
3	19FBAVe0001	2019	*Anguilla japonica*	Jul	Kidney	Gyeongnam
4	19FBAVe0002	2019	*Misgurnus mizolepis*	Jun	Kidney	Gyeongnam
5	19FBAVe0003	2019	*Carassius carassius*	Sep	Spleen	Gyeongnam
6	21FBAer0163	2018	*Cyprinus carpio nudus*	Apr	Kidney	Gyeongnam
7	21FBAer0164	2018	*Carassius carassius*	Apr	Spleen	Gyeongnam
8	21FBAer0171	2018	*Cyprinus carpio nudus*	Jun	Spleen	Gyeongnam
9	21FBAer0172	2018	*Cyprinus carpio nudus*	Jun	Kidney	Gyeongnam
10	21FBAer0174	2018	*Silurus asotus*	July	Kidney	Gyeongnam
11	21FBAer0175	2018	*Cyprinus carpio nudus*	Sep	Kidney	Gyeongnam
12	21FBAer0177	2018	*Acipenser sinensis*	Sep	Kidney	Gyeongnam
13	21FBAer0178	2018	*Anguilla japonica*	Sep	Kidney	Gyeongnam
14	21FBAer0179	2018	*Carassius carassius*	Sep	Spleen	Gyeongnam
15	21FBAer0180	2018	*Anguilla japonica*	Oct	Kidney	Gyeongnam
16	21FBAer0181	2018	*Anguilla japonica*	Oct	Kidney	Gyeongnam
17	21FBAer0182	2018	*Anguilla japonica*	Oct	Spleen	Gyeongnam
18	21FBAer0183	2018	*Anguilla japonica*	Oct	Kidney	Gyeongnam
19	21FBAer0185	2018	*Anguilla japonica*	Oct	Kidney	Gyeongnam
20	21FBAer0186	2018	*Anguilla japonica*	Oct	Spleen	Gyeongnam
21	21FBAer0187	2018	*Anguilla japonica*	Oct	Kidney	Gyeongnam
22	18FBAVe0001	2018	*Anguilla japonica*	Jan	Kidney	Gyeongnam
23	18FBAVe0002	2018	*Anguilla japonica*	Mar	Spleen	Gyeongnam
24	18FBAVe0003	2018	*Anguilla japonica*	Jun	Spleen	Gyeonggi
25	17FBAHy0005	2017	*Cyprinus carpio nudus*	May	Kidney	Gyeonggi
26	17FBAHy0010	2017	*Pelteobagrus fulvidraco*	Jul	Kidney	Gyeonggi

27	FPa4259	2011	*Carassius carassius*	May	Kidney	Gyeongbuk
28	FP3964	2010	*Cyprinus carpio nudus*	Jul	Kidney	Chungbuk
29	FP3973	2010	*Cyprinus carpio nudus*	Jul	Spleen	Chungbuk
30	FP3978	2010	*Cyprinus carpio nudus*	Jul	Spleen	Jeonnam
31	FP3980	2010	*Cyprinus carpio nudus*	Jul	Kidney	Jeonbuk
32	FPA4102	2010	*Silurus asotus*	Oct	Kidney	Gyeongbuk
33	FPA4108	2010	*Silurus asotus*	Oct	Kidney	Gyeongbuk
